# Analysis of metabolites and metabolic pathways in three maize (*Zea mays *L.) varieties from the same origin using GC–MS

**DOI:** 10.1038/s41598-020-73041-z

**Published:** 2020-10-22

**Authors:** Liyuan Zhang, Yingbo Yu, Runzhong Yu

**Affiliations:** 1 College of Food Science, Heilongjiang Bayi Agricultural University, Xinfeng road 5, Daqing, 163319 People’s Republic of China; 2 College of Electrical and Information, Heilongjiang Bayi Agricultural University, Xinfeng Road 5, Daqing, 163319 People’s Republic of China; 3 Heilongjiang Province Cultivating Collaborative Innovation Center for The Beidahuang Modern Agricultural Industry Technology, Daqing, 163319 People’s Republic of China

**Keywords:** Chemistry, Analytical chemistry, Chemical biology

## Abstract

Metabolites of the Jinyu 88, Huanong 18, and Demeiya 9 maize varieties from the same origin were isolated and identified by GC–MS, and the specific metabolites and metabolic mechanisms of these three varieties of maize were preliminarily analysed and discussed. The metabolites were extracted with 80% methanol and derived with N,O-bis(trimethylsilyl)trifluoroacetamide. A total of 59 metabolites were identified. The specific metabolites of these three varieties of maize were identified. Four possible unknown-structure metabolites were hypothesized. The results showed that the specific metabolites of JY88 were only involved in fatty acid metabolism. The specific metabolites of HN18 were determined to be involved in fatty acid metabolism, glucose metabolism, and phytosterol metabolism. The specific metabolites of DM9 were observed to participate in glucose metabolism and fatty acid metabolism. The disease resistance of HN18 was higher than that of DM9, and its grain bulk density was higher than that of DM9. JY88 was determined to be significantly different from the other two varieties, and its appearance and disease resistance were worse than those of the other two varieties. The variety with the highest nutritional value was determined to be HN18, and the variety with the lowest nutritional value was JY88. This finding indicated that different maize varieties from the same origin had different metabolites and different metabolic mechanisms, which caused the three maize varieties to exhibit different characteristics and qualities.

## Introduction

Maize (*Zea mays* L.) is the world’s most widely grown and most productive cereal crop, ranking first among the three major food crops (maize, wheat, and rice)^1^. Maize is an important food crop, feed crop and cash crop in China, and it plays an important role in China’s agricultural production^2–4^. Heilongjiang Province has the largest cultivated land area in China and is one of the three famous black lands. The three varieties of maize, that is, Jinyu 88 (JY88), Huanong 18 (HN18), and Demeiya 9 (DM9), are the main varieties of maize planted in Heilongjiang Province. The similarity of the three varieties makes them suitable for planting in the fourth cumulus temperate zone of Heilongjiang Province and is cold-resistant. However, the composition and content of different varieties of maize from the same origin may be different, since the metabolites and metabolic mechanisms produced during the growth and development of different varieties of maize are different. Maize also plays significant roles in health, blood pressure, liver protection, and fatigue recovery. All of these effects are related to maize's internal metabolites and metabolic processes. Therefore, the metabolites and metabolic mechanisms of three different varieties of maize from the same origin were studied, the natural differences in the metabolites of maize were found, the similarities and differences among different varieties of maize were summarized at the level of metabolites, and the possible reasons for these results were presented. The results of this study may provide a theoretical basis for the quality analysis of maize and provide powerful data support for further research attempting to breed and classify maize and to detect, extract, process and perform safety evaluations of the functional components of maize.

Metabolomics is the systematic study of all small-molecule endogenous metabolites with molecular weights less than 1000. Currently, gas chromatography-mass spectrometry (GC–MS) is commonly used as an analytical method in metabolomics due to its excellent chromatographic separation degree^5^, high sensitivity and high resolution. For example, Zhang et al.^6^ employed gas chromatography-mass spectrometry (GC–MS) to isolate and identify metabolites of potato tubers in northeastern China. Feng et al.^7^ studied rice metabolites from different regions of the same province by using GC/MS technology. Florent et al.^8^ successfully performed a quantitative analysis of cuticular wax in wheat tissues. Kang et al.^9^ employed GC–MS to analyse the metabolites of wheat roots and leaves with two genotypes (drought tolerance and drought sensitivity) to distinguish *Platycodon grandiflorum* and *Codonopsis pilosula* with similar morphology, Park et al.^10^ used GC–MS technology to differentiate the metabolites of the two plants and determined that there were significant differences between them. However, due to the specific properties of metabolomics, a database of standard compounds is necessary to provide the basis for data analysis. At present, there is no complete database analysis system for maize in China; therefore, further research is warranted to provide a basis for subsequent analysis.

In this work, the metabolites of three different varieties (JY88, HN18, and DM9) of maize were separated and identified by GC–MS using nontargeted metabolic profile analysis. The metabolites of the three varieties of maize were identified. The metabolic pathways of specific metabolites were investigated, and the mechanisms governing these pathways were further analysed. This research provided information for further processing of maize and elucidating the mechanisms underlying the production of metabolites. These results may also promote further improvement of the quality or other characteristics of maize through analysis of the specific metabolites of different varieties of maize and provides a basis for the classification and processing of maize or the extraction of the functional components of different maize varieties.

## Materials and methods

### Materials

The investigated varieties of conventional non-transgenic maize (*Zea mays *L.) were obtained from Zhaozhou County, which is located in Heilongjiang Province. The three varieties of maize, that is, Jinyu 88 (JY88), Huanong 18 (HN18), and Demeiya 9 (DM9), were randomly collected with a checkerboard sampling method according to the representative sampling principle within the scope of protection.

### Chemicals

Methoxyamine hydrochloride, pyridine, and N,O-bis(trimethylsilyl)trifluoroacetamide (BSTFA) were acquired from Sigma-Aldrich Co. (USA). Chromatography-grade methanol was purchased from Fisher Technologies Inc. (USA). All the aqueous solutions used in the experiment were prepared from a Milli-Q water purification system (Millipore Corp., USA) and reached the chromatographic grade standard. All other reagents of analytical grade were purchased from Beijing Chemical Factory (Beijing, China).

### Apparatus

A GC–MSQP2010 Ultra instrument (Shimadzu Technologies Inc., Japan) with an EI ion source, quadrupole mass analyser, and AOC-20i autosampler was used. The chromatographic separation of metabolites was performed on an HP-5 ms capillary column (30 m × 0.25 mm × 0.25 μm) (Agilent J&W Scientific). A Termovap Sample Concentrator (Automatic Science Instrument Co., Ltd, China), an Alpha1-2Ldplus Freeze dryer (CHRIST Co., Germany), an MSC-100 constant temperature homogenizer (Aosheng Instrument Co. Ltd., Hangzhou, China), a TGL-16B high-speed centrifuge (Anting Instrument Co. Ltd., Shanghai, China), and a KQ2200E ultrasonic cleaner (40 kHz, 100 W, Kunshan Ultrasonic Instrument Co. Ltd., Kunshan, China) were employed in this study.

### Extraction and derivatization of maize metabolites

The extraction and derivatization were performed according to the method reported by Zhang et al.^6^. Maize was crushed under the action of liquid nitrogen, sieved by a 100-mesh sieve, and stored at – 80 °C for analysis. Samples consisting of 100.00 mg maize power, 800 μL of 80% methanol solution, and 10 μL of the internal standard (2-chlorphenylalanine, 2.9 mg/mL) were placed in an Eppendorf tube (EP). The tube was vibrated for 30 s by a vortex to obtain a mixed solution. To increase the extraction efficiency, the tube containing the mixed solution was immersed into an ultrasonic bath for 9.0 min at 35 °C under a power of 80 W and shaken strongly once per minute by hand during the ultrasonic treatment. Polar metabolites were extracted from the mixed solution as follows. Subsequently, the mixed solution was centrifuged at 12,000 rpm for 10.0 min at 4 °C. After centrifugation, 200 μL liquid supernatant was transferred to a GC vial (1.5 mL autosampler vial), and then the vial was placed in the freeze dryer to dry overnight. The dry residue was completely dissolved in 30 μL of 20 mg/mL^-1^ methoxyamine hydrochloride in pyridine and incubated for 60 min at 37 °C, and 30 μL BSTFA was subsequently added to react for 60 min at 70 °C. After derivatization, the resulting solution was analysed within 24 h. Please refer to^6^ for sample processing methods.

### GC–MS analysis

The GC–MS analysis conditions were established according to the method described by Feng et al.^7^. To start the analysis, one microlitre of sample solution was injected into the chromatograph with an autosampler. The analysis of GC was performed on a 30-m HP-5 ms column with 0.25-mm inner diameter and 0.25-mm film thickness (Agilent J&W Scientific). The temperatures of the injection, interface, and ion source were set to 280 °C, 250 °C, and 230 °C, respectively. Helium was used as the carrier gas at a constant flow rate of 2 mL/min. The temperature programme was set initially at 80 °C (held for 2 min), increased at a rate of 10 °C/min to 320 °C and was set at 320 °C (held for 6 min). The system was set at 80 °C (held for 6 min) prior to injection of the next sample. Full scanning mode was used, and the scanning range was 50–550 (m/z). All sample solutions were analysed within 24 h.

### Metabolite analysis

The identification of metabolites was first performed by comparison with the NIST 14 standard database and was subsequently validated with standards measured under the same conditions. The unknown metabolites that could not be identified were recorded in the database for subsequent identity identification. The obtained information was organized, and the specific metabolites of different maize varieties were investigated.

### Metabolic pathway and mechanism analysis

The metabolic pathways of different metabolites were compared through the KEGG database (www.kegg.jp/kegg/kegg1.html). Relevant metabolic pathways were found in the various screened metabolites in KEGG, and the changes in the metabolic processes of different varieties of maize were inferred. The metabolic mechanisms of these varieties were explored and elucidated by means of enrichment analysis in Metaboanalyst and retrieval analysis of metabolic pathway in KEGG.

### Ethical standards

This article does not contain any studies with human participants or animals performed by any of the authors.

## Results and discussion

### Results of GC–MS analysis

**Figure 1:**
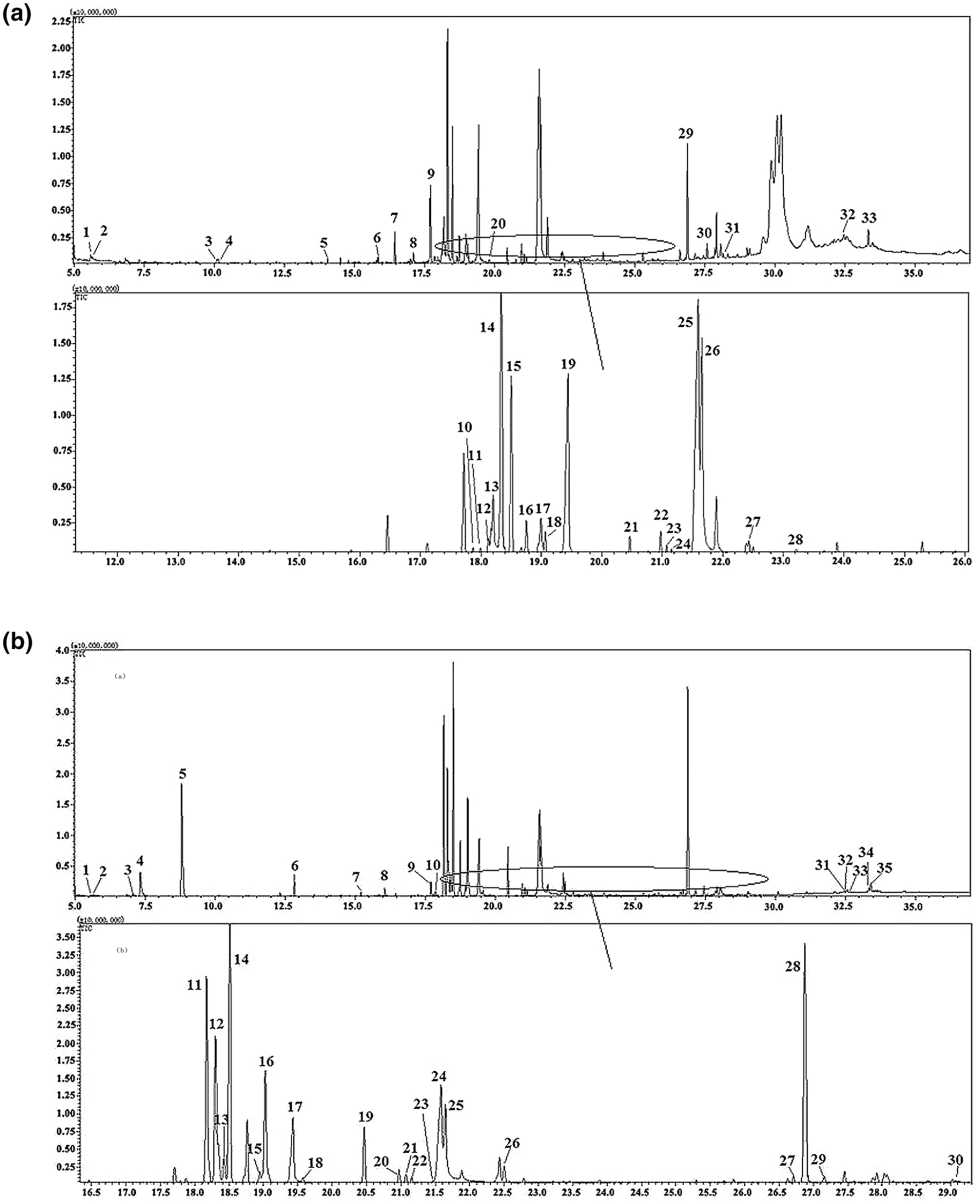

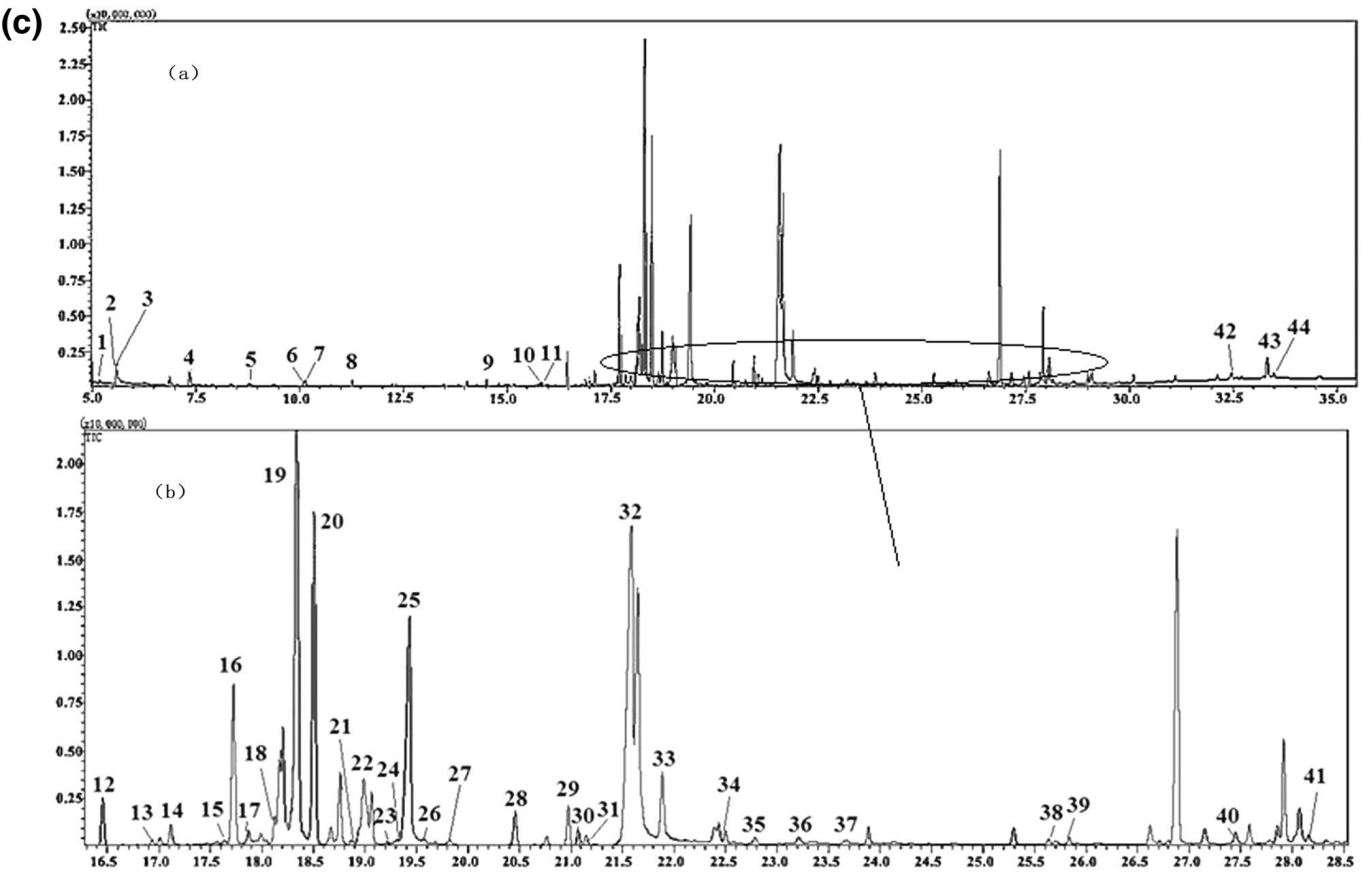
GC–MS total ion chromatogram of metabolites from maize. (1) GC–MS total ion chromatogram of metabolites from JY88 maize, (**a**) complete chromatogram of 5.00–35.00 min. (**b**) Representative expansion of the chromatogram in for the range of 12.0–26.0 min. (2) GC–MS total ion chromatogram of metabolites from HN18 maize, (**a**) complete chromatogram of 5.00–35.00 min. (**b**) Representative expansion of the chromatogram in for the range of 16.5–29.0 min. (**3**) GC–MS total ion chromatogram of metabolites from DM9 maize. (**a**) Complete chromatogram of 5.00–35.00 min. (**b**) Representative expansion of the chromatogram in for the range of 16.5–28.5 min.

The components extracted from maize were separated by gas chromatography-mass spectrometry. The total ion chromatograms of three different varieties (JY88, HN18, and DM9) of maize samples are shown in Fig. 1. It can be seen from the total ion current graphs of three maize varieties that the numbers of peaks and the intensities of the three maize varieties are different. This analysis provides abundant data and information for the characterization of metabolites. The changes in metabolites were detected by observing the signal phase intensity of the metabolites.

### Isolation and identification of metabolites

There were 33 metabolites and 3 unknown-structure metabolites detected in JY88, while 34 metabolites and 2 unknown-structure metabolites were detected in HN18. Forty-two metabolites were detected in DM9 samples, of which 5 metabolites had unknown structures. In the metabolites of the three maize varieties, a total of 59 metabolites were measured (Table 1) by GC–MS and verification with standards, while there were 5 unknown-structure metabolites, and the relative contents of the metabolites were all above 0.1%.

**Table 1:** List of metabolites in different maize.

No.	Retention time	Name	JY88	HN18	DM9
1	5.187	Propanoic acid	−	−	+
2	5.602	Unknown	+	+	+
3	5.674	Dodecane	−	+	−
4	5.685	Rhamnitol	+	−	+
5	7.338	Butane	−	+	+
6	8.794	Acetamide	−	−	+
7	8.817	Glycerol	−	+	−
8	10.095	Anethole	+	−	+
9	10.148	N-acetylindole	+	−	+
10	11.283	Tetraethylene glycol	−	−	+
11	14.056	Tagatofuranose	+	−	+
12	15.22	Arabinose	−	+	−
13	15.805	Erythritol	+	+	+
14	15.859	Phenylphosphonic acid	+	−	−
15	15.859	Unknown	−	−	+
16	16.458	Unknown	+	−	+
17	16.931	Acetin	−	+	+
18	17.116	Threose	+	−	+
19	17.719	Sorbose	+	+	+
20	17.789	Galactose	−	+	−
21	17.874	d-Xylose	+	+	−
22	17.874	d-Glucose	−	−	+
23	17.947	Tagatose	+	+	−
24	17.995	Unknown	+	−	−
25	18.125	Glucuronic acid	+	−	+
26	18.165	Fructose	+	+	+
27	18.509	Talose	+	+	+
28	18.756	Allose	+	+	−
29	18.867	Galactoside	+	+	+
30	18.944	Mannitol	−	+	−
31	19.205	Xylopyranose	−	−	+
32	19.026	d-Glucitol	−	+	+
33	19.066	Ribitol	+	−	+
34	19.31	Lyxofuranose	−	−	+
35	19.441	Ascorbic acid	+	+	+
36	19.574	Fucopyranose	−	+	−
37	19.825	Cyclopropanetetradecanoic acid, 2-Octyl-, methyl ester	−	−	+
38	20.462	Palmitic acid	+	+	+
39	20.975	Inositol	+	+	+
40	21.073	10,13-Octadecadienoic acid	+	+	+
41	21.154	7-Octadecenoic acid	+	+	+
42	21.465	Tetradecanoci acid	+	+	−
43	21.592	inoleic acid	+	+	+
44	21.648	Octadecanoic acid	+	+	+
45	22.502	13-Octadecenoic acid	+	−	−
46	23.212	α-Linolenate	+	−	+
47	23.676	Dichloroacetic acid	−	−	+
48	26.876	Sucrose	−	+	−
49	27.157	Mannobiose	+	+	+
50	27.45	Trehalose	−	−	+
51	28.158	Uridine	−	−	+
52	28.334	13-Docosenoamide	+	−	−
53	29.075	Maltose	−	+	−
54	32.465	5-Cholestene-3-ol, 24-methyl-	+	−	+
55	32.564	Campesterol	−	+	−
56	33.344	β-Sitosterol	+	+	+
57	32.46	Unknown	−	+	+
58	32.465	Stigmast-5-ene, 3β-	−	+	−
59	33.568	Ergostane	−	+	−

“Unknown” indicated the matching degree of material and database is very low.

“ + ”indicated the compound was detected. “−” indicated the compound wasn’t detected.

The known-structure metabolites could be sorted into sugars and their derivatives, fatty acids and their derivatives, alcohols, organic acids and intermediates. The primary sugars and their derivatives were tagatofuranose, arabinose, threose, sorbose, d-galactose, d-xylose, d-glucose, tagatose, fructose, talose, allose, d-galactoside, xylopyranose, lyxofuranose, fucopyranose, sucrose, mannobiose, trehalose, and maltose. The fatty acids and their derivatives were primarily palmitic acid, 10,13-octadecadienoic acid, 7-octadecenoic acid, cyclopropanetetradecanoic acid, tetradecanocic acid, linoleic acid, octadecanoic acid, 13-octadecenoic acid, and α-linolenic acid. The alcohols primarily included glycerol, rhamnitol, tetraethylene glycol, erythritol, mannitol, d-glucitol, and ribitol. The organic acids included propanoic acid, acetin, ascorbic acid, and dichloroacetic acid. The intermediates consisted of dodecane, butane, acetamide, N-acetylindole, anethole, phenylphosphonic acid, glucuronic acid, inositol, uridine, cis-13-docosenoamide, 5-cholesterene-3-ol, 24-methyl-, campesterol, β-sitosterol, stigmast-5-ene, 3β-, and ergostane. In addition, there were relatively small amounts of a number of substances, such as mannopyranoside, butanetriol, butanoic acid, deoxyribose, ribofuranose, and benzoic acid. However, these substances cannot be shown in the total ion current graphs because their relative content was less than 0.1%.

Compared with the findings of previous research^11^, the number of metabolites obtained in this work was smaller. This difference could be observed because the detection limit of this method was above the concentration of undetected metabolites, and the undetected metabolites themselves may have diversity and specificity, which warrant further research.

### Analysis of metabolic pathways and mechanisms of different maize varieties

Among the differential metabolites, there were primary metabolites^12^ and other intermediate products. Compared with the KEGG database, it was found that the specific metabolites in the experiment were primarily involved in carbohydrate metabolism, energy metabolism, and lipid metabolism. The specific metabolites selected for the maize variety are shown in Tables 2, 3 and 4.

**Table 2:** The specific metabolites of JY88.

NO	Retention time	Name
1	15.859	Phenylphosphonic acid
2	22.502	13-Octadecenoic acid
3	28.334	13-Docosenoamide

**Table 3:** The specific metabolites of HN18.

NO	Retention time	Name
1	5.674	Dodecane
2	8.817	Glycerol
3	15.22	Arabinose
4	17.789	Galactose
5	18.944	Mannitol
6	19.574	Fucopyranose
7	26.876	Sucrose
8	29.075	Maltose
9	32.564	Campesterol
10	32.465	stigmast-5-ene, 3β-
11	33.568	Ergostane

**Table 4:** The specific metabolites of DM9.

NO	Retention time	Name
1	5.187	Propanoic acid
2	8.794	Acetamide
3	11.283	Tetraethylene glycol
4	17.874	d-Glucose
5	19.205	Xylopyranose
6	19.31	Lyxofuranose
7	19.825	Cyclopropanetetradecanoic acid, 2-octyl-, methyl ester
8	23.676	Dichloroacetic acid
9	27.45	Trehalose
10	28.158	Uridine

#### Analysis of metabolic pathways and mechanisms of JY88

Table 2 shows that the specific metabolites of JY88 were determined to be phenylphosphonic acid, 13-octadecenoic acid and 13-docosadienamide. Among these metabolites, phosphorus is often present in nature in the form of phosphate and in the form of hydrogen phosphate in alkaline conditions, and the derivatization reagent was alkaline during the experiment. Phenylphosphonic acid is generally used in such materials as coordination polymers, catalysts, and heterocyclic polymers. This metabolite is a kind of chemical reagent. It is speculated that the detection of phenylphosphonic acid in maize may be attributable to the reaction of the benzene ring binding to free phosphorus and hydroxyl groups.

13-Octadecenoic acid is formed during the synthesis or decomposition of fatty acids in the acyl carrier protein. 13-Docosadienoic acid synthesis has three stages: (1) basic synthesis of the fatty acid chain (in plastids), (2) formation of oleic acid (which occurs in the endoplasmic reticulum), and (3) extension of the monounsaturated fatty acid chain (which occurs in the endoplasmic reticulum). The third phase of synthesis is based on the oleic acid in the first phase, which extends the carbon chain through the extended cycle of the superlong fatty acid chain. Each cycle can extend the carbon chain of the original fatty acid by two carbon atoms and subsequently synthesize 13-docosadienamide^13^.

#### Analysis of metabolic pathways and mechanisms of HN18

In Table 3, the specific metabolites of HN18 are presented as dodecane, glycerol, galactose, mannitol, fucose, sucrose, maltose, sitosterol, stigmasterol, and ergosterol. Specific metabolites can be primarily divided into sugars, plant sterols, and intermediates, which are primarily involved in the synthesis and decomposition of fatty acids and sugar metabolism.

Among these metabolites, dodecane accumulates during the synthesis of acyl carrier proteins or the metabolism of fatty acids. Glycerol is involved in lipid metabolism, glycolysis, glycogen heterogenesis and other pathways. After metabolism, dodecane primarily provides energy for the body and reduces the energy of the synthesis reaction. The metabolic pathway that is involved is also the final pathway of fat and protein metabolism. Galactose can be converted from galactositol, sorbitose, mannose, and glycerol by the action of enzymes. Sucrose itself is an extracellular substance that participates in the metabolism of starch and sucrose. Sucrose-6-phosphate is formed by the action of enzymes and subsequently enters the cell to start metabolism. Maltose is involved in the metabolism of starch and sucrose. Maltodextrin can be formed from starch by enzymes, maltose can be directly formed by amylases, and it can also be converted by d-6-phosphate glucose. Mannitol is involved in the metabolism of fructose and mannose, which is converted into fructose by enzymes. Phytosterol is a kind of natural active substance that widely exists in the cells and tissues of plants. Phytosterol is metabolized by the mevalonate pathway in plants. This metabolite can inhibit the absorption of cholesterol in the human body and prevent arteriosclerosis. The metabolic pathway is shown in Suppl. Figure 1. The phytosterol-mediated inhibition of the intestinal cholesterol absorption process of the synthesis mechanism has not been clearly and accurately described to date, but one possible explanation for the inhibited dissolution of cholesterol in the gut is that precipitation is formed under the action of phytosterol, and the intestine cannot absorb it. The other possible explanation is that cholesterol must contain "mixed micelles" of bile salt and lecithin, and the hydrolysis of phytosterols is easier than cholesterol. This process can reduce the solubility of cholesterol in the micelle, the age of cholesterol and its metabolite reduction in excrement and urine, which is based on the interpretation of the data^14^. Ergosterol is mainly found in fungi, such as moulds and mushrooms, and ergosterol may be detected in maize because of the presence of moulds in maize samples.

The specific metabolites of HN18 were determined primarily to be sugars and phytosterols. This kind of maize has good palatability and is easy to chew, digest and absorb. HN18 maize can reduce bile alcohol in the blood and soften blood vessels, preventing and treating coronary heart disease. HN18 maize has higher economic value.

#### Analysis of metabolic pathways and mechanisms of DM9

As shown in Table 4, the specific metabolites of this variety were determined to be propanoic acid, acetamide, tetraethylene glycol, d-glucose, xylopyranose, lyxofuranose, cyclopropanetetradecanoic acid dichloroacetic acid, trehalose, and uridine. These specific metabolites were be attributed sugars, fatty acids, organic acid, and intermediates and were determined to be primarily involved in the synthesis and decomposition of fatty acids and sugar metabolism.

Propionic acid may be formed by a fragment of 3-phosphate-d-glyceryl acyl phosphate, which is cleaved by the formation of a 3-phospho-d-glyceryl group under the action of phosphoglycerate kinase during the metabolism of starch and sugar. This metabolite may also be formed by an incomplete metabolic accumulation of propionic acid. The primary source of glucose is plant photosynthesis, which can generate energy through glycolysis and the citric acid cycle and absorb enough nutrients to provide a material source for crop metabolism to protect leaves and reduce the harm caused by pathogenic bacteria. Xylose is involved in the metabolism of ascorbic acid and alginate, the interconversion of pentose and glucuronate, and the metabolism of amino sugars and nucleosides. Xylose can be formed by the formation of gulonic acid by UDP-glucose or myo-inositol by the action of enzymes or by the metabolism of arabinose. Xylose is the unit of the sugar chain linked to serine (or threonine) in certain glycoproteins. The free state of xylose has not been observed in nature^15^. Natural d-xylose is found in plants as a polysaccharide. Lysucrose is involved in the process of interconversion between pentose and glucuronide, which is metabolized by xylulose. Trehalose^16^ by two glucose molecules by hemiacetal hydroxyl condensation of the reducing disaccharides. This sugar is involved in the metabolism of starch and sucrose and is formed by UDP glucose through trehalose-6-phosphate synthase. Dichloroacetic acid is observed because pesticide residues in the soil are degraded and formed by microbes. The trehalose that is produced can be transformed with the action of maltose by enzymes. Uridine is a nucleotide, and its full name is uracil ribonucleotide. Uridine is a synthetic material of RNA. Studies have found that the addition of 3′-uridylate (U) to eukaryotic RNA may be a very common and conserved phenomenon^17^. Currently, many human diseases have been found to be related to RNA uridylation, such as cancer^18^ or cardiac myotonic dystrophy^19^. In the process of fatty acid synthesis oxidation and pyruvate formation of acetyl CoA, acetyl groups may be present. It is possible that acetamide is an intermediate in its metabolic pathway or a combination of an acetyl group and a free amino group. The dichloroacetic acid was detected due to the presence of chlorine in the experimental reagent, and the acetic acid could be converted by acetyl CoA or by acetaldehyde. It is speculated that tetraethylene glycol is a derivative of ethanol.

When metabolites are involved in the metabolic pathway, the final substances to be expressed cause maize varieties to be different. For example, glycerol in HN18 is involved in the triacylglycerol biosynthesis pathway, and triacylglycerol is an important component of lipoprotein and plays an important role in metabolism as a vehicle for energy and fat in food. Triacylglycerol has twice the energy density of sugar and protein, and its content is also related to arteriosclerosis^20^.

The specific metabolites of DM9 can be attributed to sugars, fatty acids, and organic acids. This kind of maize has better quality and higher yield than the other two varieties.

As shown in Tables 2 and 4, different maize varieties have specific metabolites. Although three maize varieties were derived from the same origin, they had their own characteristics, and their metabolic pathways and mechanisms were still different. Among the three varieties, HN18 is the main variety that is resistant to disease and had a large grain capacity. Through a comparative analysis of the three varieties of maize, it was found that the difference between DM9 and HN18 was mainly in phytosterols. It is speculated that the difference in plant sterols caused HN18 to be more resistant to disease than DM9, and the grain bulk density was higher than that of DM9. JY88 was most different from the other two varieties; therefore, its appearance and disease resistance were worse than those of the other two varieties. It was also found that HN18 had the highest nutritional value, while JY88 had the lowest nutritional value. In the face of changes in the same environment, the three varieties of maize exhibited different metabolic mechanisms and responses with different metabolites, and the nutrition and value of maize also changed, which may also be attributable to different gene expression profiles in different maize varieties^21^.

### Identification of unknown metabolite structure

**Figure 2:**
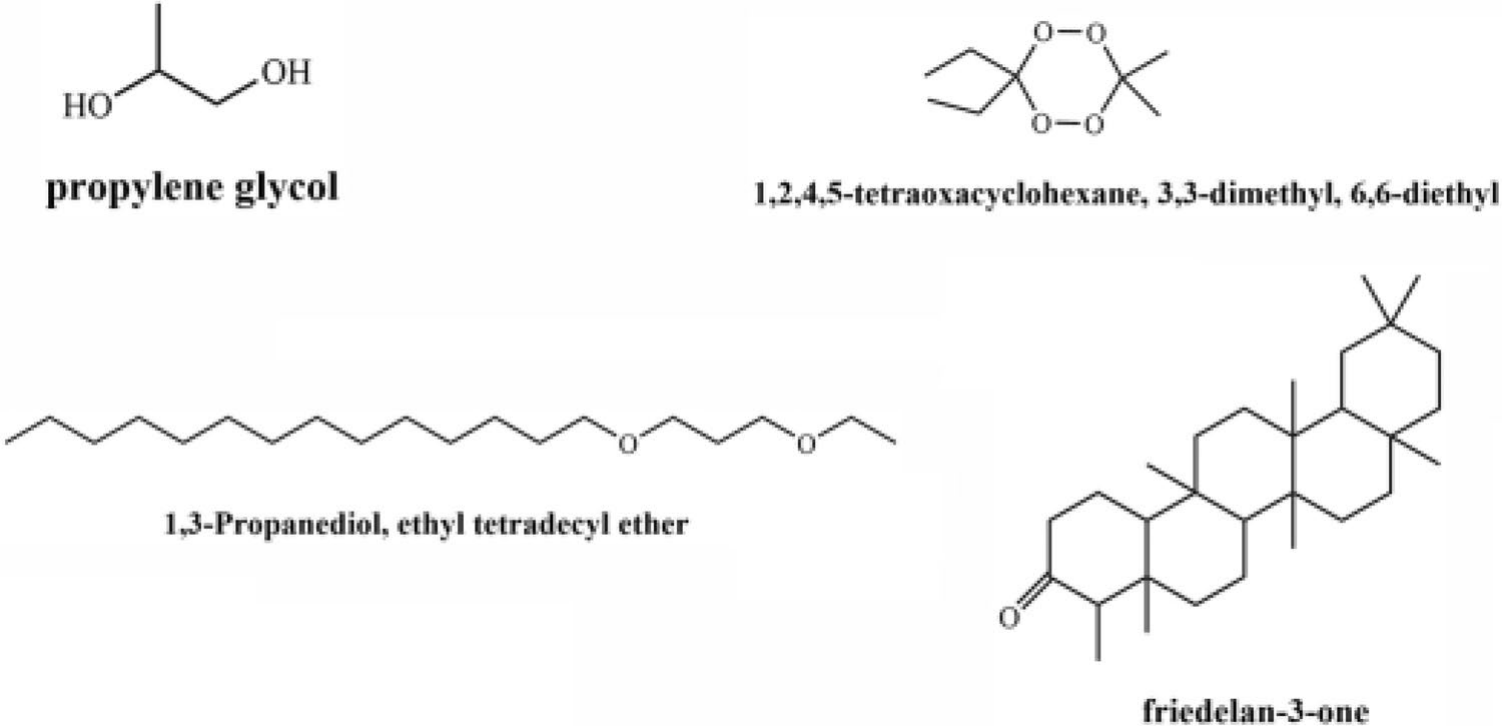
Speculate the possible structure of unknown compound.

Based on the principle of silanization, it is speculated that the silyl group introduces and replaces the active hydrogen, thereby reducing the polarity of the compound and reducing the binding of hydrogen bonds, and the derivatives formed are volatile. C_6_H_16_O_2_Si has been speculated to be propylene glycol, C_18_H_40_O_2_Si has been speculated to be 1,3-propanediol and ethyl tetradecyl ether, C_13_H_32_O_4_Si_2_ has been speculated to be 1,2,4,5-tetraoxacyclohexane, 3,3-dimethyl, and 6,6-diethyl, and C_33_H_58_OSi has been speculated to be friedelan-3-one; the structural formula is shown in Fig. 2.

Propylene glycol is the intermediate product in the process of glycerol metabolism. Propylene glycol is metabolized by hydroxyacetone in the metabolic process. Propylene glycol is further metabolized into propane through the action of enzymes and is interconverted with l-propanal. The presence of 1,3-propylene glycol may be attributable to the conversion of glycerol by microorganisms^22^. The existence of 1,3-propanediol and ethyl tetradecyl ether may be due to the hydrogen bond between tetradecanoic acid oxidation and 1,3 propylene glycol. 1,2,4,5-tetraoxacyclohexane is a natural peroxide found in plants. As an important secondary metabolite of plant synthesis, friedelan-3-one is ubiquitous on the surface of organ tissue^23^ and plays a protective role.

## Conclusions

In this work, the metabolomics of three different varieties of maize (*Zea mays *L.) were developed by using nontargeted metabolic profile analysis based on GC–MS, and the metabolites in maize (*Zea mays *L.) were separated and identified. It was found that there are three specific metabolites (fatty acids) in JY88, which are primarily involved in fat metabolism. Eleven specific metabolites of HN18 were found; among these 11 metabolites, sugars were the most abundant followed by plant sterol, and there was only one fatty acid and one intermediate product. The metabolic pathway of plant sterols was found in the KEGG database, and it was also involved in the metabolism of fat and sugar. The ten specific metabolites of DM9 were found, among which sugars were the most abundant followed by fatty acids, intermediate products, and organic acids. These metabolites participate in the metabolism of fats and sugars. HN18 is resistant to disease and has a large grain capacity. HN18 is more resistant to disease than DM9, and the grain bulk density is higher than DM9. JY88 has worse properties than the other two varieties. HN18 has the highest nutritional value, while JY88 has the lowest. These results showed that the metabolites and metabolic mechanisms of the three maize varieties were different, which resulted in the different characteristics that were expressed by the three maize varieties. The different characteristics exhibited by different varieties of maize may be related to the diversity and contents of different metabolites.

## Supplementary information

Supplementary file 1

Supplementary file 2

## Data Availability

The all data used to support the findings of this study are available from the corresponding author upon request.
