# Supplementary material for: Analysis of metabolites and metabolic pathways in three maize (Zea mays L.) varieties from the same origin using GC–MS
Source: Sci Rep. 2020 Oct 22;10:17990. doi: 10.1038/s41598-020-73041-z (PMC7581747; doi:10.1038/s41598-020-73041-z)
Supplement: Supplementary file 1 [file 41598_2020_73041_MOESM1_ESM.docx]

@article{Li2001Current,

title={Current Situation and Development Countermeasures of Corn Seed Processing Industry in China},

author={Li-Sheng, L. I. and Pan, Shi Qiang and Zhang, Sheng Wen},

journal={Journal of Jilin Agricultural University},

year={2001},

}

@article{Yu2008Current,

title={Current Status of Corn Germplasm Resources Innovation in China},

author={Yu-Sheng, Y. E. and Sun, Jia and Hao, Nan and Zhe, L. I.},

journal={Seed},

volume={8},

number={1},

pages={1-25},

year={2008},

}

@article{Kyung2016Anti,

title={Anti-inflammatory effects of Zea mays L. husk extracts.},

author={Kyung-Baeg and Roh and Hyoyoung and Kim and Seungwoo and Shin and Young-Soo and Jung-A and Lee and Mi and },

journal={BMC complementary and alternative medicine},

year={2016},

}

@article{Shao2019Recent,

title={Recent advances and perspectives of metabolomics-based investigations in Parkinson’s disease},

author={Shao, Yaping and Le, Weidong},

journal={Molecular Neurodegeneration},

volume={14},

number={1},

year={2019},

}

@article{ShackletonGas,

title={Gas chromatography/mass spectrometry (GC/MS) remains a pre-eminent discovery tool in clinical steroid investigations even in the era of fast liquid chromatography tandem mass spectrometry (LC/MS/MS)},

author={Shackleton, Cedric H. L.},

journal={J Steroid Biochem Mol Biol},

volume={121},

number={3-5},

pages={496-504},

}

@article{article,

author = {Zhang, Liyuan and Yu, Yingbo and Wang, Changyuan and Zhang, Dongjie},

year = {2018},

month = {08},

pages = {},

title = {Isolation and Identification of Metabolites in Chinese Northeast Potato (Solanum tuberosum L.) Tubers Using Gas Chromatography-Mass Spectrometry},

volume = {12},

journal = {Food Analytical Methods},

doi = {10.1007/s12161-018-1336-5}

}

@article{Yuchao2019Research,

title={Research on Differential Metabolites in Distinction of Rice ( Oryza sativa L . ) Origin Based on GC-MS},

author={Yuchao Feng and TianXin Fu and Liyuan Zhang and Changyuan Wang and Dongjie Zhang},

year={2019},

}

@article{Florent2018GC,

title={GC-MS Metabolomics to Evaluate the Composition of Plant Cuticular Waxes for Four Triticum aestivum Cultivars},

author={Florent, Lavergne and Corey, Broeckling and Darren, Cockrell and Scott, Haley and Frank, Peairs and Courtney, Jahn and Adam, Heuberger},

journal={International Journal of Molecular Sciences},

volume={19},

number={2},

pages={249-},

year={2018},

}

@article{Zhiyu2019Comparative,

title={Comparative metabolomic profiling in the roots and leaves in contrasting genotypes reveals complex mechanisms involved in post-anthesis drought tolerance in wheat.},

author={Zhiyu and Kang and Md and Ali and Babar and Naeem and Khan and Jia and Guo and Jahangir and },

journal={Plos One},

year={2019},

}

@article{Park2019Discrimination,

title={Discrimination of Platycodon grandiflorum and Codonopsis lanceolata using gas chromatography-mass spectrometry-based metabolomics approach},

author={Park, Hyeon-Yong and Shin, Jeoung-Hwa and Boo, Hee-Ock and Gorinstein, Shela and Ahn, Yun Gyong},

journal={Talanta},

volume={192},

pages={486-491},

year={2019},

}

@article{Harrigan2016Evaluation,

title={Evaluation of metabolomics profiles of grain from maize hybrids derived from near-isogenic GM positive and negative segregant inbreds demonstrates that observed differences cannot be attributed unequivocally to the GM trait},

author={Harrigan, George G. and Venkatesh, Tyamagondlu V. and Leibman, Mark and Blankenship, Jonathan and Perez, Timothy and Halls, Steven and Chassy, Alexander W. and Fiehn, Oliver and Xu, Yun and Goodacre, Royston},

year={2016},

}

@article{Situ2001Secondary,

title={Secondary metabolites: metabolic pathways, classification, action and production (Ⅰ)},

author={Situ, L.L. & Yuan, C.Y.},

year={2001},

}

@article{Tian2015The,

title={The Biosynthesis and Metabolic Engineering of Very Long-chain Monounsaturated Fatty Acid},

author={Tian, Deyu and Wang, Shian and Wang, Lihao and Wang, Jialin and Li, Fuli},

journal={Biotechnology Bulletin},

year={2015},

}

@article{Liu2018The,

title={The Lipid-lowering Function of Plant Sterols and Stanols},

author={Liu Xinlong and Chen Guanghui and Zhao Rui and Hang Zhongxia},

journal={modern food},

year={2018},

}

@article{Zhang2010A,

title={A study on the consecutive preparation of d-xylose and pure superfine silica from rice husk},

author={Zhang, Hongxi and Xu, Zhao and Ding, Xuefeng and Hong, Lei and Xue, Chen and An, Dongmin and Li, Yunling and Wang, Zichen},

journal={Bioresource Technology},

volume={101},

number={4},

pages={1263-1267},

year={2010},

}

@article{Griffiths2016Metabolite,

title={Metabolite transport and associated sugar signalling systems underpinning source/ sink interactions},

author={Griffiths, Cara A and Paul, Matthew J and Foyer, Christine H},

journal={Biochimica Et Biophysica Acta},

volume={1857},

number={10},

pages={1715-1725},

year={2016},

}

@article{Hagan2009Lin28,

author = {Hagan, J. P. and Piskounova, E. and Gregory, R. I.},

title = {Lin28 recruits the TUTase Zcchc11 to inhibit let-7 maturation in mouse embryonic stem cells},

journal = {Nat Struct Mol Biol},

volume = {16},

number = {10},

pages = {1021-5},

year = {2009},

}

@article{Treiber2012Regulation,

title={Regulation of microRNA biogenesis and function},

author={Treiber, Thomas and Treiber, Nora and Meister, Gunter},

journal={Thromb Haemost},

volume={107},

number={4},

pages={605-610},

year={2012},

}

@article{YAO2018Relation,

title={Relation between triglyceride and high density lipoprotein cholesterol ratios with progression of arterial stiffness in normotensive subjects},

author={YAO Tao and TIAN Bolin and HU Dan and LI Gang and LIU Zhang and Department of Neurology},

journal={the journal of practical medicine},

year={2018},

}

@article{LIU2018Determination,

title={Determination of free sugars in Rhizoma Polygonati Odorati by silanization GC/MS},

author={LIU Ying-jiao and XIAO Lan and LUO Lin-min and GONG Li-min and LIU Ta-si and CHEN Nai-hong},

journal={Journal of International Pharmaceutical Research},

year={2018},

}

@article{Skogerson2010Impact,

author = {Skogerson, Kirsten and Harrigan, George G. and Reynolds, Tracey L. and Halls, Steven C. and Ruebelt, Martin and Iandolino, Alberto and Pandravada, Anand and Glenn, Kevin C. and Fiehn, Oliver},

title = {Impact of Genetics and Environment on the Metabolite Composition of Maize Grain},

journal = {Journal of Agricultural and Food Chemistry},

volume = {58},

number = {6},

pages = {3600-3610},

year = {2010}

}

@article{Chotani2011Enzyme,

title={Enzyme production in culture medium comprising raw glycerol},

author={Chotani, Gopal K. and Herfert, Kenneth F. and Reimann, Janine},

year={2011},

}

@article{Momo2011ANTIMICROBIAL,

title={ANTIMICROBIAL ACTIVITY OF THE METHANOLIC EXTRACT AND COMPOUNDS FROM THE STEM BARK OF GARCINIA LUCIDA VESQUE (CLUSIACEAE).},

author={Momo and Itbert and Joseph and Kuete and Victor and Dufat and Hanh and Michel and Sylvie and Wandji and },

journal={International Journal of Pharmacy & Pharmaceutical Sciences},

year={2011},

}
