# Supplementary figures and images for: Analysis of metabolites and metabolic pathways in three maize (Zea mays L.) varieties from the same origin using GC–MS
Source: Sci Rep. 2020 Oct 22;10:17990. doi: 10.1038/s41598-020-73041-z (PMC7581747; doi:10.1038/s41598-020-73041-z)

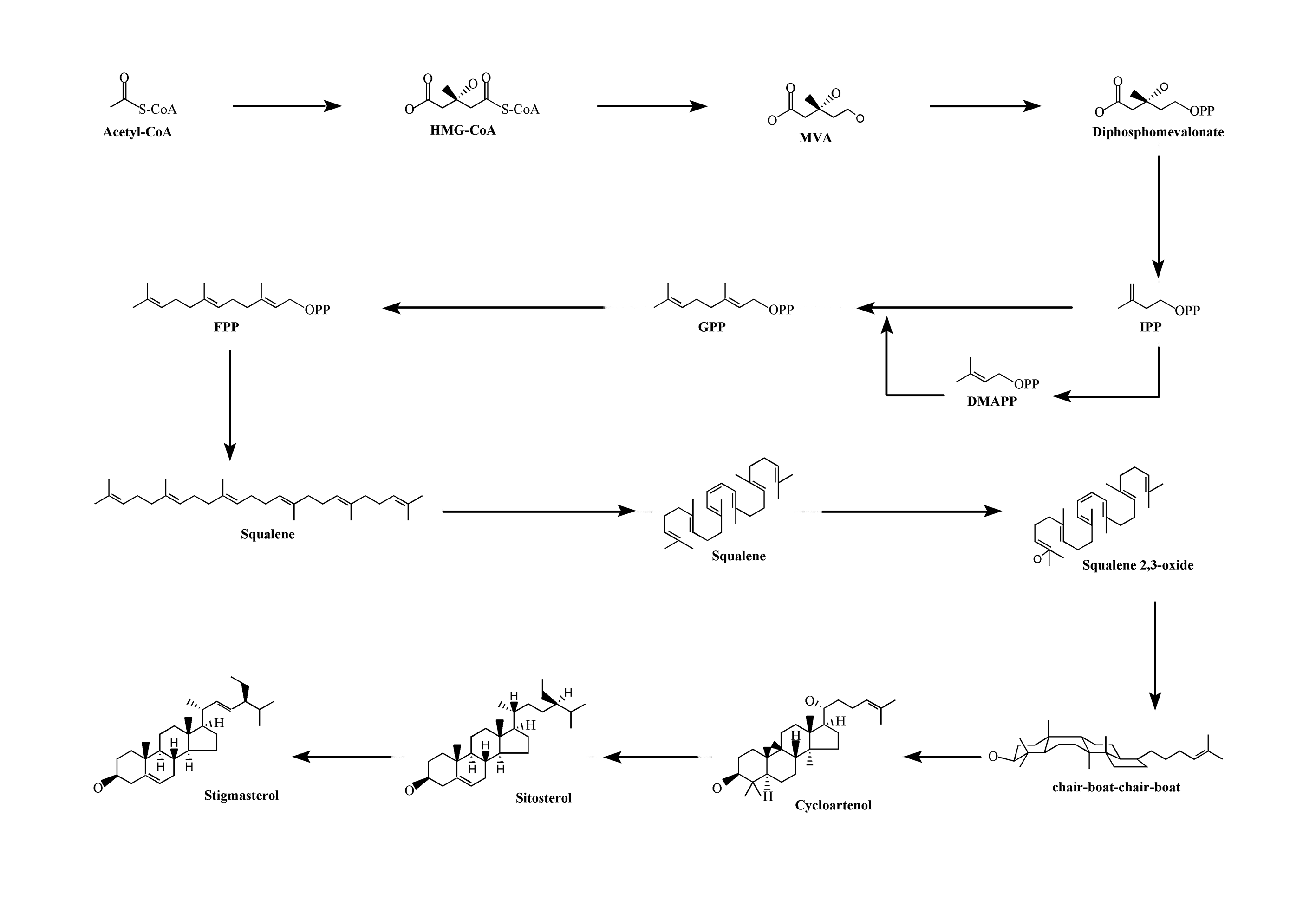


**Suppl. Fig. 1 Metabolic pathway of sterols in maize**

Supplement: Supplementary file 2 [file 41598_2020_73041_MOESM2_ESM.doc]
